# Global rice multiclass segmentation dataset (RiceSEG): comprehensive and diverse high-resolution RGB-annotated images for the development and benchmarking of rice segmentation algorithms

**DOI:** 10.1016/j.plaphe.2025.100099

**Published:** 2025-09-04

**Authors:** Junchi Zhou, Haozhou Wang, Yoichiro Kato, Tejasri Nampally, P. Rajalakshmi, M. Balram, Keisuke Katsura, Hao Lu, Yue Mu, Wanneng Yang, Yangmingrui Gao, Feng Xiao, Hongtao Chen, Yuhao Chen, Wenjuan Li, Jingwen Wang, Fenghua Yu, Jian Zhou, Wensheng Wang, Xiaochun Hu, Yuanzhu Yang, Yanfeng Ding, Wei Guo, Shouyang Liu

**Affiliations:** a Engineering Research Center of Plant Phenotyping, Ministry of Education, Jiangsu Collaborative Innovation Center for Modern Crop Production, Sanya Institute of Nanjing Agricultural University, Academy for Advanced Interdisciplinary Studies, Nanjing Agricultural University, Nanjing, China; b Graduate School of Agricultural and Life Sciences, The University of Tokyo, Tokyo, Japan; c Department of Artificial Intelligence, Indian Institute of Technology, Hyderabad, India; d Department of Electrical Engineering, Indian Institute of Technology, Hyderabad, India; e Institute of Biotechnology, Professor Jayashankar Telangana Agricultural State University, Hyderabad, India; f Graduate School of Agriculture, Kyoto University, Kyoto, Japan; g Key Laboratory of Image Processing and Intelligent Control, School of Artificial Intelligence and Automation, Huazhong University of Science and Technology, Wuhan, China; h National Key Laboratory of Crop Genetic Improvement, National Center of Plant Gene Research, and Hubei Key Laboratory of Agricultural Bioinformatics, Huazhong Agricultural University, Wuhan, China; i State Key Laboratory of Efficient Utilization of Arid and Semiarid Arable Land in Northern China, The Institute of Agricultural Resources and Regional Planning, Chinese Academy of Agricultural Sciences, Beijing, China; j Center for Geospatial Information, Shenzhen Institutes of Advanced Technology, Chinese Academy of Science, Shenzhen, China; k School of Information and Electrical Engineering, Shenyang Agricultural University, Shenyang, China; l Rice Research Institute, Jilin Academy of Agricultural Sciences, Changchun, China; m Institute of Crop Sciences/National Key Facility for Crop Gene Resources and Genetic Improvement, Chinese Academy of Agricultural Sciences, Beijing, China; n Yuan Long Ping High-Tech Agriculture Co., Ltd., Changsha, China

**Keywords:** RiceSEG dataset, Rice phenotyping, Semantic segmentation, Deep learning, Crop monitoring

## Abstract

The development of computer vision-based rice phenotyping techniques is crucial for precision field management and accelerated breeding, which facilitate continuously advancing rice production. Among phenotyping tasks, distinguishing image components is a key prerequisite for characterizing plant growth and development at the organ scale, enabling deeper insights into ecophysiological processes. However, owing to the fine structure of rice organs and complex illumination within the canopy, this task remains highly challenging, underscoring the need for a high-quality training dataset. Such datasets are scarce, both because of a lack of large, representative collections of rice field images and because of the time-intensive nature of the annotation. To address this gap, we created the first comprehensive multiclass rice semantic segmentation dataset, RiceSEG. We gathered nearly 50,000 high-resolution, ground-based images from five major rice-growing countries (China, Japan, India, the Philippines, and Tanzania), encompassing more than 6000 genotypes across all growth stages. From these original images, 3078 representative samples were selected and annotated with six classes (background, green vegetation, senescent vegetation, panicle, weeds, and duckweed) to form the RiceSEG dataset. Notably, the subdataset from China spans all major genotypes and rice-growing environments from northeastern to southern regions. Both state-of-the-art convolutional neural networks and transformer-based semantic segmentation models were used as baselines. While these models perform reasonably well in segmenting background and green vegetation, they face difficulties during the reproductive stage, when canopy structures are more complex and when multiple classes are involved. These findings highlight the importance of our dataset for developing specialized segmentation models for rice and other crops. The RiceSEG dataset is publicly available at www.global-rice.com.

## 1 Introduction

As a core pillar of global agricultural production, rice is widely cultivated worldwide and feeds more than half of the global population [1]. However, facing global warming, the variability and uncertainty in rice-growing environments pose severe challenges for the sustainability of rice production [2]. To leverage unfavorable growth conditions, great efforts have been made to improve both cultivar and cultivation practices according to the adaptation of phenotypic traits [3]. Hence, the success of these efforts strongly relies on the precision and throughput of the plant phenotyping techniques. Unfortunately, the measurement of plant phenotypic traits is accomplished mainly manually, which is very time-consuming and labor-intensive [4,5,6]. Therefore, the development of high-throughput phenotyping techniques is crucial for overcoming these limitations and consequently ensuring rice production [7,8,9].

Compared with traditional human observation, computer vision techniques have greatly advanced plant phenotyping because they provide higher throughput and accuracy [10]. A key step in this domain is image segmentation, which underpins the extraction of critical traits such as canopy structure [11], light interception [12], and stress status [13]. With respect to single-class segmentation, when green vegetation is distinguished from the background, deep learning-based models have demonstrated robust performance across various crops [14,15], maintaining consistent accuracy under diverse environments, genotypes, and spatial resolutions [16]. However, there is a growing need for more detailed segmentation that distinguishes multiple plant organs (e.g., panicles and both green and senescent leaves), as this enables deeper insights into organ development and the source–sink relationship [17]. Moreover, because weeds commonly appear in rice fields, simultaneously segmenting weeds alongside crop organs both reduces misclassification and informs weed management strategies. Although recent deep learning segmentation models, such as the SAM [18], show promise, none have successfully addressed the multiclass segmentation of rice canopies, encompassing both organs and weeds, across diverse genotypes and environmental conditions. This is primarily due to the unique challenges posed by rice canopies, which feature fine leaves, thin stems, and substantial genotype-dependent variations. Fluctuating field illumination further complicates segmentation by creating mutual shading within the canopy, while reflective water surfaces in paddy fields produce mirror-like reflections and glare, distorting certain image regions and reducing clarity. As with other complex computer vision tasks, improving current models or developing specialized approaches hinges on the availability of comprehensive training datasets that capture the full complexity of rice field conditions.

High-quality training datasets are critical for adapting state-of-the-art computer vision (CV) models to plant phenotyping [19]. In recent years, numerous phenotyping datasets have emerged for various crops, both indoors and in the field, focusing primarily on plant counting [20], organ detection [21,22], and disease or pest classification [23,24]. However, few datasets target semantic segmentation because of the labor-intensive nature of pixel-level annotation. This issue is especially pronounced in rice, where fine leaves and dense canopies complicate the annotation process, leading to a shortage of publicly available datasets [25,26]. Table 1 provides an overview of representative plant semantic segmentation datasets, which for rice crops are largely confined to single classes—such as panicle segmentation [27] for basic green segmentation from the background [28]. In summary, no existing rice segmentation dataset jointly encompasses multiple genotypes, diverse field conditions, multiple organs (leaf, stem, and panicle), and weeds.

**Table 1:** Representative semantic segmentation datasets.

Dataset	Crop type	Class	# Images	Image Size
CVPPP [29]	Rosette plants	2	1311	2048 ​× ​2448
CWFID [30]	Carrot	3	60	1291 ​× ​966
Oil Radish Growth [31]	Oil radish	7	129	1601 ​× ​1601
PhenoBench [32]	Sugar beet	3	2872	1024 ​× ​1024
Paddy Rice Imagery [27]	Rice	2	400	4096 ​× ​2160
VegAnn [28]	Rice, wheat, etc.	2	466	512 ​× ​512

RiceSEG	Rice	6	3078	512 ​× ​512

The main objectives of this work are to construct a broad, multiclass, high-resolution semantic segmentation dataset for rice crops. This dataset includes 3078 ground-based RGB images collected from 5 countries and 12 different institutions, taken throughout the entire growth cycle, and covering a wide range of genotype‒environment‒management combinations. Pixels in all the images are finely annotated into six categories: background, green vegetation, senescent vegetation, panicle, weed, and duckweed. Furthermore, to assess the dataset, we report baseline results for most classic and cutting-edge semantic segmentation algorithms. The main contributions of this study are twofold:

• To the best of our knowledge, we present the largest global rice semantic segmentation dataset, offering precise pixel-level annotations across multiple detailed classes in real rice fields.
• We conducted extensive experiments with various segmentation models on this dataset to create benchmark performance, thereby facilitating the development of more effective rice segmentation algorithms.

## 2 Materials and methods

### 2.1 Dataset collection

To maximize the representativeness and diversity of the dataset, we collected approximately 50,000 images in total, contributed by 12 institutions between 2012 and 2024, from 14 sites located in 5 countries, including China, Japan, India, the Philippines, and Tanzania (Table 2). They were taken by different types of cameras, such as digital single-lens reflex cameras, portable action cameras, or smartphones. The configuration of the cameras was set 1–2 ​m above the canopy with different orientations (0°–90^o^) toward the canopy. This ensured the high resolution of the images with ground sampling distances (GSD) ranging from 0.1 to 1.8 mm/pixel.

1) **Dataset from China.** The dataset originated from various sites across China, encompassing all major rice production regions from the northeasternmost to the southernmost areas where rice is cultivated. This extensive coverage includes more than 6000 rice varieties, resulting in a large collection of diverse images. Specifically, images provided by Nanjing Agricultural University (JS_1, JS_2, JS_3, JS_4, and HN) were meticulously gathered from experimental fields in Jiangsu and Hainan Provinces, featuring more than one thousand rice varieties. These images highlight the challenges associated with segregating plant organs due to the high variability in canopy structures among genotypes under diverse field light conditions, as well as the presence of weeds or duckweed in the background. Additionally, images from Changsha were captured in the rice experimental fields of Yuan Long Ping High-Tech Agriculture Co., Ltd. (https://lpht.com.cn/), a leading firm in rice breeding renowned for its hybrid rice varieties. This collection includes images of nearly 5000 rice genotypes at various growth stages (transition and reproductive stages), encompassing both domestic and international varieties.

**Table 2:** Metadata of the subdatasets comprising the RiceSEG dataset.

Name		Institute	Site	Images	Lat (°)	Long (°)	Year	Growth stage^a^	Genotypes	Platform	Camera	Image size (pixels)	GSD (mm/px)
CHINA	JS_1	NJAU	Jiangsu	4000	31.5 ​N	119.3 ​E	2020	Vegetative, Transition	1000	Handheld rod	SONY RX0	4800∗3200	0.1–0.3
	JS_2			4000			2021	Vegetative					
	JS_3			8000			2023	Vegetative, Transition					
	JS_4			8000			2023	Reproductive					
	HN		Hainan	2000	18.2 ​N	109.5 ​E	2023	Vegetative, Transition					0.3–0.5
	GX	HUST	Guangxi	280	24.3 ​N	109.4 ​E	2012	Vegetative	20	Fixed rod	Canon EOS 1100D	4272∗2848	0.3&1.2
	JX		Jiangxi	355	28.7 ​N	115.9 ​E	2013	Vegetative	35	Fixed rod	OLYMPUS E−450	3648∗2736	1.8
	HB	HZAU	Hubei	104	30.5 ​N	114.3 ​E	2016	Transition	104	Tripod	NIKON D7100	6000∗4000	0.3
	HLJ	CAAS	Harbin	40	45.7 ​N	126.6 ​E	2016	Vegetative	40	Handheld rod	NIKON D7100	2000∗2000	0.6
	GD	CAS	Guangdong	90	22.6 ​N	113.1 ​E	2022	Reproductive	60	Handheld rod	iphone11	2048∗1536	0.1–0.3
	LN	SYAU	Shenyang	154	41.8 ​N	123.4 ​E	2024	Vegetative, Transition	50	Handheld rod	SONY RX0	4032∗3024	0.1–0.3
	HUN	LPHT	Changsha	14994	28.2 ​N	112.9 ​E	2024	Transition	5000	Handheld rod	SONY RX0	4800∗3200	0.1–0.3
	JL	JAAS	Changchun	2642	43.8 ​N	125.3 ​E	2024	Reproductive	700	Handheld rod	SONY RX0	4800∗3200	0.1–0.3
JAPAN	TKO_1	UTokyo	Tokyo	645	35.4 ​N	139.3 ​E	2013	Vegetative	5	Fixed rod	Canon EOS Kiss x5	5184∗3456	0.1
	TKO_2			142			2014	All stage					
	TKO_3			768			2015	Transition					
INDIA	TG	IITH	Telangana	271	17.3 ​N	78.4 ​E	2018	Vegetative	50	Handheld rod	Sony RX100	5472∗3648	0.3–0.5
TANZANIA	Kil	KATC	Kilimanjaro	126	3.45 ​S	37.4 ​E	2019	Reproductive	4	Handheld rod	RICOH WG-4	3072∗2304	0.2–0.4
PHILIPPINES	Lag	IRRI	Laguna	200	14.2 ​N	121.2 ​E	2014	Vegetative	1596	Handheld rod	OLYMPUS TG-620	1600∗1200	0.3–0.5

a Growth stages are categorized into three main phases: (a) vegetative: seedling, tillering, and jointing; (b) transition: shooting, heading, and flowering; and (c) productive: filling and maturity.

The northeastern region significantly contributes to China's production of rice, particularly high-quality japonica rice adapted to cold climates. Images were collected from each of the northeastern provinces, including Heilongjiang (HL), Jilin (JL), and Liaoning (LN). The ‘HL’ dataset was captured by the Institute of Agricultural Resources and Regional Planning using a fisheye camera, providing a unique wide-angle perspective of the rice canopy across several varieties. The ‘JL’ dataset comprises images from more than 700 rice varieties obtained from the Rice Research Institute of the Jilin Academy of Agricultural Sciences, whereas the ‘LN’ dataset was provided by Shenyang Agricultural University. The ‘JX’ and ‘GX’ subdatasets, contributed by Huazhong University of Science and Technology, document images from various growth stages ranging from seedling to jointing across more than 40 genotypes in Jiangxi and Guangxi Provinces, respectively. The ‘HB’ subdataset, provided by Huazhong Agricultural University, includes data from 104 varieties, and the ‘GD’ dataset, supplied by the Shenzhen Institute of Advanced Technology, Chinese Academy of Sciences, encompasses images from more than 60 genotypes.

2) **Dataset from Japan.** This dataset encompasses a broad spectrum of rice genotypes in Japan. Notably, the dataset sourced from the University of Tokyo (TKO_1, TKO_2, TKO_3) comprises time series images of rice captured by field-fixed cameras. The UTokyo dataset was collected from paddy phenotyping field trials at the Institute for Sustainable Agro-ecosystem Services (ISAS) (35°44′20.3″N, 139°32′29.8″E) in Tokyo, Japan, during the 2014 season. A field server system cite{utokyo_2015} collected images of five genotypes throughout the entire growth stage. The camera module of the system is based on a digital single-lens reflex (DSLR) camera, the Canon EOS Kiss X5 camera, with an EF-S18-55 ​mm lens (Canon, Inc., Tokyo) that provides high-quality and high-resolution (18 megapixels) image data. A preprogrammed microcontroller board automatically controls the power and shutter of the camera.
3) **Dataset from India.** The dataset was obtained from the Institute of Biotechnology of Professor Jayashankar Telangana State Agriculture University, which is located in Hyderabad, Telangana, India. The study area covers an area of 15.3 ​m ​× ​34.8 ​m and includes two repetitions of 203 plots, each representing a different variety/genotype of aerobic paddy, resulting in a total of 406 plots. Each plot covers an area of 1.26 square meters and contains 42 crop strands. The dataset provides a collection of images of upland rice, which are unique because of the presence of many weeds in complex backgrounds. The images were captured by a team from the Indian Institute of Technology Hyderabad using a high-resolution Sony RX 100 camera. Each image has a resolution of 3456 ​× ​2592 pixels.
4) **Dataset from the Philippines.** The dataset was collected from the International Rice Research Institute (IRRI) farm located in Los Baños, Philippines at 14°11 ​N, 121°15 ​E and an elevation of 21 ​m above sea level. The study encompassed three distinct paddy fields containing a comprehensive collection of rice varieties with varying experimental conditions. In total, the dataset comprises 1596 rice varieties/lines distributed across 2172 plots, with some overlap in varieties between fields. All the fields maintained a consistent planting density of 20 ​cm ​× ​20 ​cm between plants, creating a uniform growing environment for comparative analysis. The experimental design allows for the systematic evaluation of rice phenotypes under different field management strategies. All the images were captured during the vegetative stage of rice growth, specifically 3–4 weeks after transplanting.
5) **Dataset from Tanzania.** Field experiments were conducted at the irrigated lowland field in the Kilimanjaro Agricultural Training Centre in the Republic of Tanzania (3°45′08″ S, 37°39′68″ E, 720 ​m above sea level) in 2019. Four rice varieties, NERICA 1, IR64, TXD 306 and Wahiwahi, were subjected to four different water management practices with three replications: continuous flooded conditions and three alternate wetting and drying conditions. Irrigation was repeated until the water depth reached 10 ​cm when the surface water level decreased to 0 ​cm, 15 ​cm and 30 ​cm, respectively. At maturity, images of the rice canopy were taken vertically downward from 80 ​cm above the rice canopy using a digital camera (WG-4; Ricoh, Japan). Twenty-four rice hills (4 hills ​× ​6 hills, 1.2 ​m ​× ​0.9 ​m) that were captured in the images were then harvested to ground level, and the yield and yield components were investigated.

### 2.2 Construction of the RiceSEG dataset

**Fig. 1:**
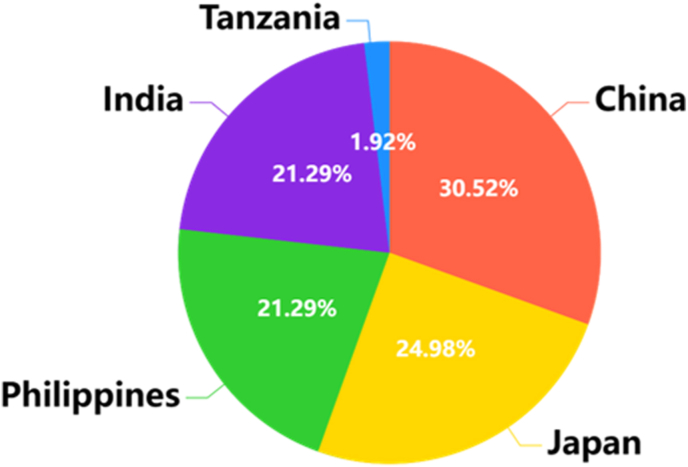
Global range distribution and composition of the datasets (a detailed station distribution map is shown in Fig. S1).

**Fig. 2:**
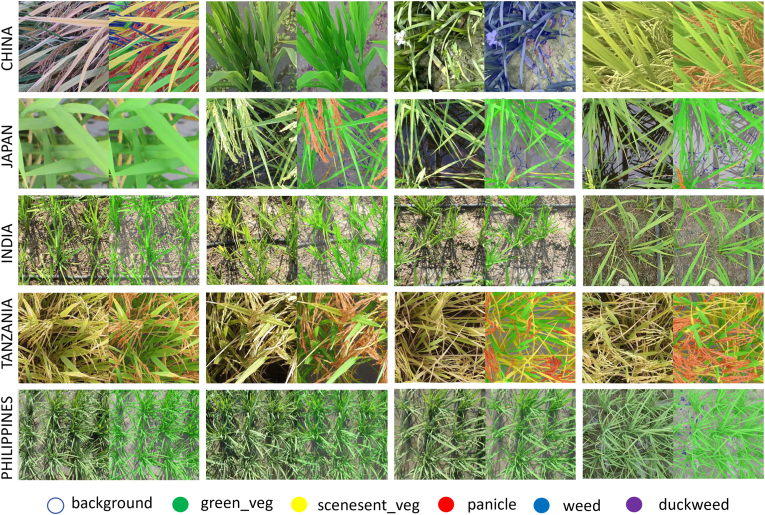
Image annotation process.

Considering the substantial variation in the number of images collected from China and other countries (Table 2 and Fig. 1), we employed distinct selection strategies to maximize the dataset's representativeness (Fig. 2 and Table 3). In China, compared with other countries, collaborations across all major rice-growing regions enabled the largest overall collection of images. From each Chinese site, 60–100 images were randomly chosen to capture diverse growth stages, varieties, and environmental conditions. In contrast, acquiring high-resolution rice images from other countries proved more challenging; hence, for the remaining five countries, we utilized nearly all the originally collected data.

**Table 3:** Statistics of the RiceSEG dataset.

Name	Images	No. of classes	Category Proportions (%)					
			background	green_veg	senescent_veg	panicle	weed	duckweed
JS_1	100	5	47.1	50.6	0.2	0.5		1.6
JS_2	100	4	53.9	44.4	0.2			1.6
JS_3	100	6	25.3	22.9	0.4	0.4	36.2	14.9
JS_4	80	5	11.4	37.1	32.7	16.4	2.5	
HN	100	5	56.8	42.6	0.2		0.1	0.3
GX	60	3	79.0	19.0	1.9			
JX	60	2	76.8	23.2				
HB	100	4	24.9	67.2	5.6	2.4		
HLJ	100	2	48.6	51.5				
GD	100	4	8.3	72.5	4	15.1		
LN	60	6	21.5	70.7	3.6	1.6	0.2	2.5
HUN	100	5	3.0	66.4	4.5	26.1	0.1	
JL	60	5	6.4	63.2	1.2	26.4		2.8
TKO_1	100	5	49.6	47.6	2.1	2.2	0.7	
TKO_2	504	6	67.4	28	0.8	2.3	0.8	0.8
TKO_3	100	4	15.4	82.3	2.1	0.2		
TG	600	5	58.9	39.1	1.2	0.2	0.6	
Kilimanjaro	54	4	15.7	27.5	25.8	30.9		
Laguna	600	4	55.7	43.5	0.3		0.3	

**Summary 3078**	**3078**	**6**	**48.3**	**43.4**	**2.5**	**3.4**	**1.6**	**0.8**

Cropping can increase the relative size of small but important targets (e.g., panicles, senescent leaves, and weeds) and capture phenotypic variation within the same scene, thereby enhancing organ-scale feature learning and dataset diversity. Most datasets adopted this approach, such as GWHD [21,22], VegAnn [28], and PhenoBench [32]. After finalizing the image selection across all the sites, a cropping procedure was adopted. With continual advancements in computational resources, larger models can leverage higher-resolution images for potentially enhanced performance [33]. Nonetheless, balancing the annotation costs with the demand for high-resolution imagery led us to fix the final cropping size to 512 ​× ​512 pixels. For the Chinese dataset, we selected a representative 512 ​× ​512 subimage from each original image, whereas for images from other countries, a 1024 ​× ​1024 region was first cropped from the center and then subdivided into one to four subimages using a sliding-window approach, with each subimage carefully inspected for quality.

### 2.3 Data annotation

We engaged specially trained volunteers, primarily graduate students studying agronomy at Nanjing Agricultural University, to manually annotate the images. In total, the annotation process involved 11 volunteers, with the time cost for each image ranging from 0.5 to 1.5 ​h, depending on its complexity. Collectively, the annotators dedicated 2440 ​h to data annotation and an additional 800 ​h to verification and refinement, culminating in a total of 3240 ​h.

The training program included fundamental knowledge of rice growth physiology, equipping annotators to identify diverse characteristics and morphological traits of rice at various growth stages. Participants were further trained to categorize each pixel into one of six predefined classes: background, green vegetation, senescent vegetation, panicle, weed, and duckweed, which were labeled as numbers from 0 to 5, respectively. Detailed explanations and annotation samples of each category were provided to ensure consistent classification criteria (Fig. 2). Moreover, annotators were trained to use a JavaScript-based image annotation tool (https://github.com/kyamagu/js-segment-annotator) [34]. This tool was selected because it was developed on the basis of the superpixel annotation method. This approach significantly enhances annotation efficiency while ensuring precise alignment with natural boundaries. Note that annotators were required to adjust the superpixel resolution carefully to capture fine details and textures in the rice images.

To ensure annotation quality and consistency, a strict protocol was followed throughout the process. After the initial round of annotation, approximately 10 ​% of the labeled images from each annotator were randomly selected for double-checking by a second annotator. During this process, common misclassifications were identified and corrected, with documentation provided by the project leader. Feedback was then promptly given to the annotators to improve their practices. In summarizing the lessons learned from this iterative annotation process, we found that among the six categories, distinguishing senescent leaves, particularly those at the bottom of the canopy with substantial shadows, was often challenging. Additionally, residual plant matter from previous crop rotations sometimes resembled senescent rice, further complicating the labeling task. To minimize subjectivity, each annotation was cross-verified by at least three individuals to ensure reliability. Finally, weeds such as water onions, which structurally resemble rice at certain growth stages, were sometimes misclassified as green vegetation. Extra care was taken to maintain precision in the annotations.

Owing to the nature of agricultural ecosystems, the labels in the RiceSEG dataset are not evenly distributed across categories, as expected (Table 3). The background category is the most dominant, accounting for nearly 50 ​% of all labels. Following this, the green vegetation category ('green_veg’) ranks second, comprising more than 40 ​%, as green plants cover a significant portion of the rice fields and are the primary visual component throughout the growth cycle. In contrast, categories such as senescent vegetation ('senescent_veg’) and rice panicle ('panicle’) appear only during the reproductive stage and thus represent a relatively small proportion of the dataset. Additionally, owing to the use of herbicides across all the experimental sites, the presence of weeds and duckweed is minimal. We released an additional file, class_pixel_counts.csv, which lists the per-image pixel count of all six classes to facilitate customized subset creation.

### 2.4 Baseline test

#### 2.4.1 Baseline models

To create the baseline accuracy for the RiceSEG dataset, we determined baseline results for six semantic segmentation models divided into two major categories (Table 4): Convolutional neural networks (CNN) and transformer-based models. We chose the FCN [35,36], PSPNet [37], and DeepLabV3+ [38], which are three methods based on the CNN backbone. With respect to the Transformer architecture, we adopted SegFormer [39], KNet [40] and Mask2Former [41]. These models represent the classic and cutting-edge technologies in the field of semantic segmentation. Our RiceSEG dataset was randomly split 8:2 for the training and test datasets. All six models selected were trained and tested accordingly.

**Table 4:** Baseline model for semantic segmentation.

Model		Backbone	Venue	Key Features
CNN backbone	FCN	Resnet50	2015-CVPR/2017-TPAMI [35,36]	Fully convolutional network for semantic segmentation
	PSPNet		2017-CVPR [37]	Employing pyramid pooling to capture multiscale contextual information.
	DeepLabV3+		2018-ECCV [38]	Combining atrous convolutions with a new decoder for enhanced boundary delineation.
Transformer backbone	SegFormer	Mit b0	2021-NeurIPS [39]	Efficient transformer-based model with a lightweight MLP decoder.
	KNet	SwinT	2021-NeurIPS [40]	Uses kernel-based convolution for multiscale feature extraction.
	Mask2Former		2022-CVPR [41]	Unifies semantic and instance segmentation with dynamic mask prediction.

#### 2.4.2 Evaluation metrics

At the pixel scale, to evaluate the baseline models, we determined the intersection over union (IoU) and accuracy for each class while using the mean intersection over union (mIoU) and mean accuracy (mAcc) as performance metrics across all classes. Afterward, at the image scale, we calculated the proportion of each class in the entire image and compared it with the corresponding proportions from the manually labeled images. Furthermore, we calculated R^2^ and RMSE values to assess the model's performance.

## 3 Results

### 3.1 Dataset diversity analysis

**Fig. 3:**
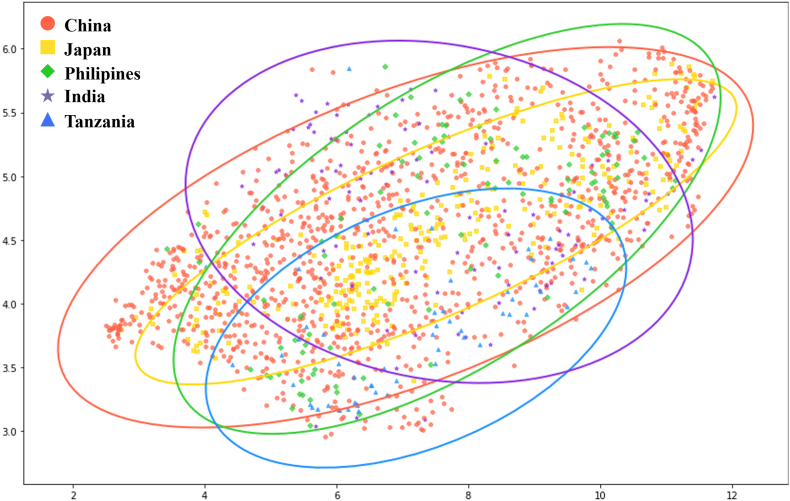
Distribution of subdatasets from different contributions. The images were projected into two dimensions using uniform manifold approximation and projection (UMAP). Each colored domain represents the confidence ellipse (90 ​%) of the country's dataset. The color of each ellipse matches the points of its respective country.

The UMAP projections of the RiceSEG samples from the five contributing countries are shown in Fig. 3. Overall, the data from China exhibit a relatively larger distribution area because of the broad variation in genotype–environment–management factors in the collected rice images. In contrast, the distribution of samples from the other four countries largely overlaps with that of the Chinese dataset but within a narrower domain. Nevertheless, the datasets from these four countries demonstrate distinct distribution patterns. Ultimately, the combined samples from all five countries contribute to expanding the dataset's distribution and improving its representation of the diverse range of high-resolution field images of rice.

### 3.2 Baseline results at the pixel scale

**Fig. 4:**
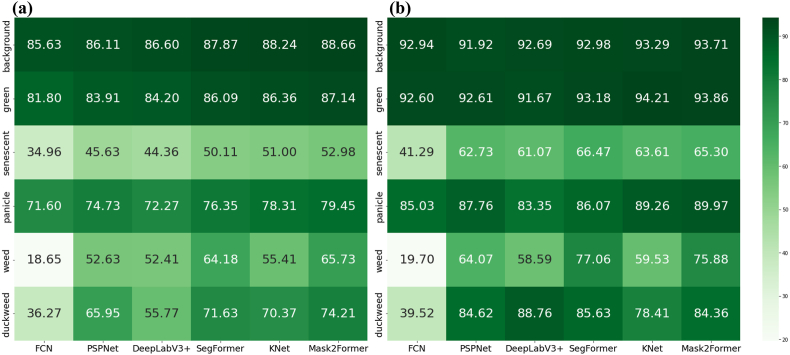
Segmentation performance at the pixel scale. Six classical and state-of-the-art semantic segmentation models were compared in terms of the IOU (a) and ACC (b). The test dataset includes 601 images in 6 classes.

With respect to the average performance across all the classes, the transformer-based segmentation models outperformed their CNN counterparts (Table 5). Specifically, all the baseline models generally performed well in segmenting background, green vegetation, and panicles. However, significant differences were observed in more challenging categories, such as senescent vegetation, weeds, and duckweed. With respect to senescent vegetation, none of the models yielded satisfactory results; the best-performing model, Mask2Former, achieved an IoU of only 52.98, and SegFormer achieved an ACC of 66.47. For weeds, although the classification accuracy of the top-performing model reached 77.06, the IoU remained low at 65.73 (Fig. 4).

**Table 5:** Performances of different models on the RiceSEG dataset.

Metrics	CNN backbone			Transformer backbone		
	FCN	PSPNet	DeepLabv3+	SegFormer	KNet	Mask2Former
mIoU	54.82	68.16	65.93	72.70	71.87	**74.69**
mAcc	61.85	80.48	79.35	83.57	80.50	**83.85**

**Fig. 5:**
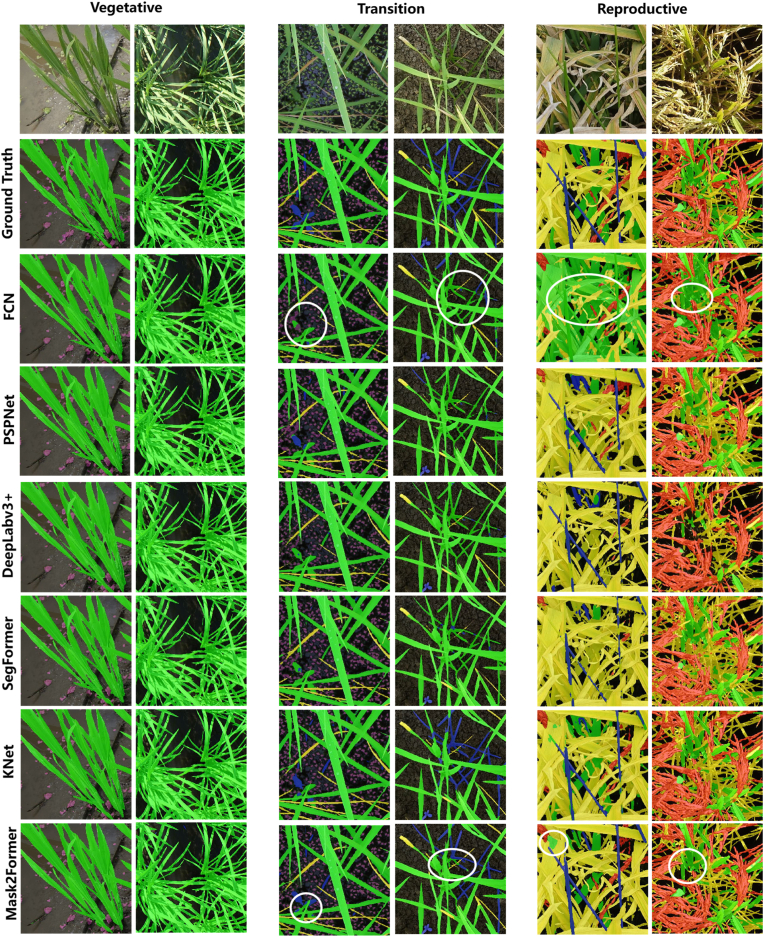
Visualization of the segmentation results on the test set. Six classical and state-of-the-art semantic segmentation models were tested on three growth stages of rice. The white circles represent key misclassified regions.

The segmentation performance of all the baseline models on the test set is shown in Fig. 5. During the vegetative growth stages, the majority of the images consist of green vegetation and background, which are highly contrasted and easily distinguishable. As a result, only minor differences in segmentation performance were observed among the models during this phase. However, during the transition phase, segmentation became more challenging because of the emergence of weeds and duckweed. The high morphological similarity between these and rice parts leads to misidentification as rice, resulting in less accurate segmentation and an increased occurrence of false positives. In the reproductive stage, the canopy begins to saturate, leaving only a small portion of the background visible. This leads to the misclassification of yellow leaves, which are predominantly classified as green vegetation or background. Achieving reliable recognition performance remains difficult for both traditional CNN models and state-of-the-art transformer models.

### 3.3 Baseline results at the imaging scale

**Fig. 6:**
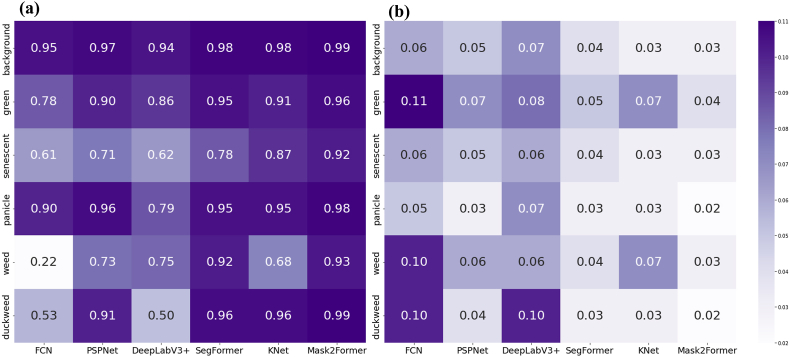
Segmentation performance at the image scale. Six classical and state-of-the-art semantic segmentation models were compared in terms of R2 (a) and RMSE (b) values. The test dataset includes 601 images in 6 classes. The vertical axis corresponds to the proportion of each class's pixels relative to the total pixels in the entire image.

**Fig. 7:**
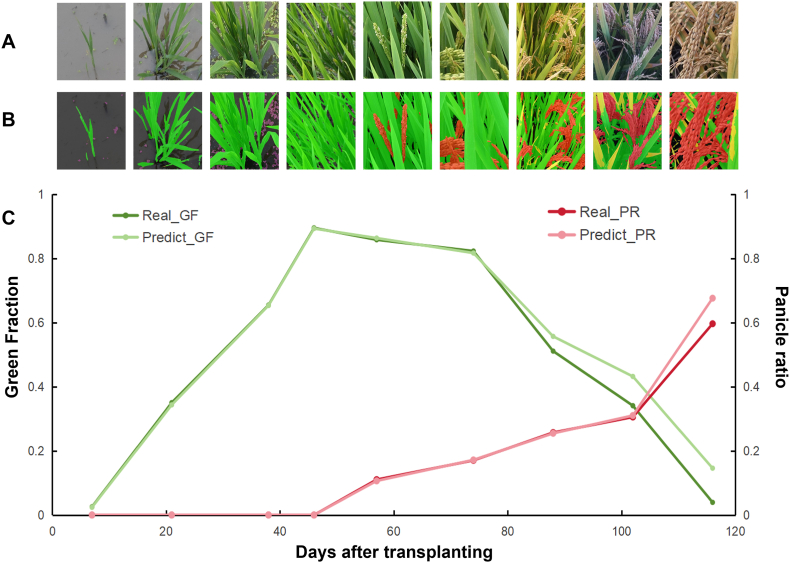
Dynamics of the GF and leaf-to-panicle ratio generated by time series images. (A) RGB images and (B) estimated (C) dynamics of the GF and panicle ratio.

At the image scale, for green vegetation and panicles, the models generally performed well. However, for more complex categories, such as weeds and senescent vegetation, the CNN models performed poorly. In contrast, transformer-based models significantly improved performance (Fig. 6). Furthermore, we demonstrated the dynamics of the rice canopy from the seedling stage to the maturity stage on the basis of the best-performing Mask2Former model (Fig. 7). This further indicates that dispersals of the segmentation at the reproductive stage are consistent with those at the pixel scale.

## 4 Discussion

### 4.1 Potential contribution of the RiceSEG dataset

To the best of our knowledge, we created the first comprehensive multiclass rice semantic segmentation dataset, RiceSEG. We gathered nearly 50,000 high-resolution, ground-based images from five major rice-growing countries (China, Japan, India, the Philippines, and Tanzania), encompassing more than 6000 genotypes across all growth stages. From these original images, 3078 representative samples were selected to form the RiceSEG dataset. Notably, the subdataset from China spans all major genotypes and rice-growing environments from northeastern to southern regions. The RiceSEG uniquely captures key rice crop organs, including the primary source organs—leaves (classified as green and senescent)—and the sink organ, the panicle. Unlike previous rice segmentation datasets, which were limited to binary segmentation of vegetation and background [14], our dataset enables the development of advanced segmentation models to track the dynamics of these critical organs throughout the entire rice growth cycle (Fig. 6). Providing detailed time series data on organ development offers insights that are unattainable through manual measurements, potentially revealing new ecophysiological processes underlying crop adaptation to local environments and yield formation [42]. Additionally, the dataset incorporates both aquatic and nonaquatic weeds, enabling simultaneous segmentation of weeds and rice crops. By facilitating accurate weed and crop differentiation, datasets may play a crucial role in the development of advanced computer vision models for automated weed control, addressing the growing demand for precision agriculture solutions such as field robots [43]. However, existing models encounter difficulties during the reproductive stage, when canopy structures become more complex and multiple classes are involved. These findings highlight the importance of our dataset for developing specialized segmentation models for rice and other crops. Finally, through collaboration with international partners, we expanded the dataset to include samples from 5 countries, representing diverse genotype–environment–management combinations. This broad representation ensures the robustness and scalability of the resulting segmentation models, enabling precise differentiation of fine phenotypic traits among hundreds or even thousands of genotypes for breeding programs.

### 4.2 Challenges in rice image segmentation

Compared with other computer vision tasks, semantic segmentation in agriculture—particularly for rice—presents unique challenges. In the broader computer vision field, widely used datasets such as COCO and ADE20K typically encompass a larger number of categories and significantly more images than RiceSEG. However, these general-purpose datasets predominantly feature large objects with relatively planar surfaces, whereas crop images often contain dense, finely detailed structures—primarily leaves—characterized by numerous edges and complex spatial arrangements. This inherent complexity is further compounded by varying illumination within the canopy, where mutual shading and reduced light transmittance at greater canopy depths make it particularly difficult to segment leaves located near the bottom. In paddy rice fields, water surfaces introduce additional complications, including reflections and mirror-like effects that resemble vegetation, whereas submerged or partially submerged weeds add yet another layer of segmentation difficulty. Although a few existing datasets address crop image segmentation, their limited scope and categories do not fully capture the complexity of real-world agricultural settings. Consequently, our RiceSEG dataset offers distinct value for developing and validating specialized segmentation models tailored to rice and other plant species.

Because crop image segmentation datasets are both scarce and unique, current state-of-the-art methods are not fully optimized because of the complexities inherent in rice imagery. Nonetheless, owing to the robust feature-extraction capabilities of deep learning models, most tested architectures accurately classify dominant image components (e.g., background and green vegetation) at the pixel level. Beyond pixel-level performance, we also evaluated segmentation accuracy at the image scale, as many phenotypic trait estimations (e.g., the green vegetation fraction for the green area index, [11]) depend on organ-specific pixel fractions. Overall, image-level evaluations largely parallel pixel-level results but exhibit slight improvements, potentially because of compositional effects across each image. However, pronounced performance gaps remain in more challenging categories, such as senescent vegetation, weeds, and duckweed. Transformer-based models (e.g., SegFormer and Mask2Former) demonstrate superiority in these domains, likely because their self-attention mechanisms capture long-distance dependencies and effectively handle intricate visual patterns [44]. In contrast, CNN-based architectures, which primarily extract local features, struggle to recognize fine structures that require a more global contextual understanding [45]. Moving forward, research could focus on further refining transformer-based models to enhance segmentation performance in these nuanced categories.

### 4.3 Limitations of the dataset

We made significant efforts to collect rice images from the most representative rice-growing conditions. Nevertheless, our dataset still has limitations regarding its overall representativeness. For instance, in China, we gathered images from nearly all major rice-producing regions, capturing a wide range of genotype–environment–management combinations. In contrast, although we obtained an almost equivalent number of images in Japan, the Philippines, and India, their geographic and genotypic diversity is much narrower, potentially biasing the model toward Chinese conditions and reducing its generalizability elsewhere. Additionally, in assembling each site's dataset, we included images spanning all growth stages to improve the model's ability to handle the entire crop cycle. Despite this, the annotated pixel counts across categories are imbalanced, particularly for senescent leaves, which constitute only 2.8 ​% of the annotated pixels. This imbalance may partly account for the relatively low segmentation accuracy observed for senescent leaves (Fig. 5). However, for images collected in natural environments, such a pixel distribution is a normal representation of the natural world. Another factor could be the inherent ambiguity of annotating senescent leaves, especially those in lower canopy layers where shading is more pronounced. Furthermore, our current dataset does not include a detailed classification of weeds. To achieve more precise in-field weed management, a broader range of weed species is essential. Therefore, we are considering both collecting more field data and employing data generation techniques to further diversify the dataset. Previous studies [[46], [47], [48], [49], [50]] have demonstrated the potential of synthetic data: by supplementing a small set of real images and labels with high-quality virtual samples, it is possible to approach the desired accuracy while greatly reducing annotation costs. Additionally, foundation models [51] trained on large-scale unsupervised data have shown strong performance across a wide range of downstream tasks—even when only small amounts of labeled data are available.

To facilitate distribution and track updates, we provide detailed descriptions of the dataset at http://www.global-rice.com and http://www.phenix-lab.com. Unfortunately, open-access datasets remain scarce in plant phenotyping research. In contrast, the computer vision community has achieved rapid progress largely through shared resources that reduce redundant efforts and enhance efficiency. We encourage more researchers in plant phenotyping and digital agriculture to collectively foster an open-access culture. Such collaboration will expedite the development of robust deep learning algorithms for agricultural applications, ultimately having a greater impact on crop breeding and smart farming.

## Authors’ contributions

Conceptualization, S.L., J.Z., and W.G.; Methodology, S.L., and W.G.; Software, J.Z., and H.W.; Validation, J.Z.; Formal analysis, S.L., J.Z., and W.G.; Investigation, J.Z., Y.G. and F.X.; Data curation, W.G., Y.K., T.N., M.B., P.R., K.K., H.L., Y.M., H.C., Y.C., W.Y., W.L., J.W., F.Y., J.Z., X.H., Y.Y. and W.W.; Writing original draft preparation, J.Z.; Writing review and editing, J.Z., S.L., and W.G.; and Supervision, S.L. and W.G.

## Funding

This work was supported by the National Key R&D Program of China (No. 2022YFE0116200and No. 2022YFD2300700), the Young Scientists Fund of the 10.13039/501100001809National Natural Science Foundation of China (No. 42201437and No. 32201893), the PhD Scientific Research and Innovation Foundation of The 10.13039/501100010834Education Department of Hainan Province Joint Project of 10.13039/100020760Sanya Yazhou Bay Science and Technology City (No. HSPHDSRF-2024-09-001), the Hainan Provincial Natural Science Foundation of China (No. 325QN370), the “JBGS” Project of Seed Industry Revitalization in Jiangsu Province (JBGS [2021] 007), the Japan Society for the Promotion of Science (No. 22KK0083and No. JP25H01110), and the Sarabetsu Village “Endowed Chair for Field Phenomics” project in Hokkaido, Japan.

## Data availability

The RiceSEG dataset is publicly available at http://www.global-rice.com/.

## Conflicts of interest

The authors declare that there are no conflicts of interest relevant to the content of this article.

## References

[1] Jin Z., Shah T., Zhang L., Liu H., Peng S., Nie L. (2020). Effect of straw returning on soil organic carbon in rice–wheat rotation system: a review. Food Energy Secur..

[2] Godfray H.C.J., Beddington J.R., Crute I.R., Haddad L., Lawrence D., Muir J.F., Pretty J., Robinson S., Thomas S.M., Toulmin C. (2010). Food security: the challenge of feeding 9 billion people. Science.

[3] Cassman K.G., Harwood R.R. (1995). The nature of agricultural systems: food security and environmental balance. Food Policy.

[4] Chen C., Mcnairn H. (2006). A neural network integrated approach for rice crop monitoring. Int. J. Rem. Sens..

[5] Madec S., Jin X., Lu H., De Solan B., Liu S., Duyme F., Heritier E., Baret F. (2019). Ear density estimation from high resolution RGB imagery using deep learning technique. Agric. For. Meteorol..

[6] Mandal D., Kumar V., Bhattacharya A., Rao Y.S., Siqueira P., Bera S. (2018). Sen4Rice: a processing chain for differentiating early and late transplanted rice using time-series Sentinel-1 SAR data with google Earth engine. IEEE Geosci. Remote Sens. Lett..

[7] Maohua W. (2001). Possible adoption of precision agriculture for developing countries at the threshold of the new millennium. Comput. Electron. Agric..

[8] Mermut A.R., Eswaran H. (2001). Some major developments in soil science since the mid-1960s. Geoderma.

[9] Yandun Narvaez F., Reina G., Torres-Torriti M., Kantor G., Cheein F.A. (2017). A survey of ranging and imaging techniques for precision agriculture phenotyping. IEEE ASME Trans. Mechatron..

[10] Li Z., Guo R., Li M., Chen Y., Li G. (2020). A review of computer vision technologies for plant phenotyping. Comput. Electron. Agric..

[11] Wang J., Lopez-Lozano R., Weiss M., Buis S., Li W., Zhang J., Baret F. (2020).

[12] Shouyang L., Shichao J., Qinghua G., Yan Z., Fred B. (2020). An algorithm for estimating field wheat canopy light interception based on digital plant phenotyping Platform. Smart Agric..

[13] Baret F., Madec S., Irfan K., Lopez J., Comar A., Hemmerlé M., Dutartre D., Praud S., Tixier M.H. (2018). Leaf-rolling in maize crops: from leaf scoring to canopy-level measurements for phenotyping. J. Exp. Bot..

[14] Gao Y., Li Y., Jiang R., Zhan X., Lu H., Guo W., Yang W., Ding Y., Liu S. (2023). Enhancing green fraction estimation in rice and wheat crops: a self-supervised deep learning semantic segmentation approach. Plant Phenomics.

[15] Serouart M., Madec S., David E., Velumani K., Lopez Lozano R., Weiss M., Baret F. (2022). SegVeg: segmenting RGB images into green and senescent vegetation by combining deep and shallow methods. Plant Phenomics.

[16] Gao Y., Li L., Weiss M., Guo W., Shi M., Lu H., Jiang R., Ding Y., Nampally T., Rajalakshmi P., Baret F., Liu S. (2024). Bridging real and simulated data for cross-spatial- resolution vegetation segmentation with application to rice crops. ISPRS J. Photogrammetry Remote Sens..

[17] Zhao Z., Wang C., Yu X., Tian Y., Wang W., Zhang Y., Bai W., Yang N., Zhang T., Zheng H., Wang Q., Lu J., Lei D., He X., Chen K., Gao J., Liu X., Liu S., Jiang L., Wan J. (2022). Auxin regulates source-sink carbohydrate partitioning and reproductive organ development in rice. Proc. Natl. Acad. Sci..

[18] Kirillov A., Mintun E., Ravi N., Mao H., Rolland C., Gustafson L., Xiao T., Whitehead S., Berg A.C., Lo W.-Y., Dollár P., Girshick R. (2023). *Segment anything* (arXiv:2304.02643). arXiv.

[19] Garcia-Garcia A., Orts-Escolano S., Oprea S., Villena-Martinez V., Garcia-Rodriguez J. (2017, April 22). A review on deep learning techniques applied to semantic segmentation. arXiv.Org.

[20] Bai X., Liu P., Cao Z., Lu H., Xiong H., Yang A., Cai Z., Wang J., Yao J. (2023). Rice plant counting, locating, and sizing method based on high-throughput UAV RGB images. Plant Phenomics.

[21] David E., Madec S., Sadeghi-Tehran P., Aasen H., Zheng B., Liu S., Kirchgessner N., Ishikawa G., Nagasawa K., Badhon M.A., Pozniak C., de Solan B., Hund A., Chapman S.C., Baret F., Stavness I., Guo W. (2020). Global Wheat Head Detection (GWHD) dataset: a large and diverse dataset of high-resolution RGB-Labelled images to develop and benchmark wheat head detection methods. Plant Phenomics.

[22] David E., Serouart M., Smith D., Madec S., Velumani K., Liu S., Wang X., Pinto F., Shafiee S., Tahir I.S.A., Tsujimoto H., Nasuda S., Zheng B., Kirchgessner N., Aasen H., Hund A., Sadhegi-Tehran P., Nagasawa K., Ishikawa G., Guo W. (2021). Global wheat head detection 2021: an improved dataset for benchmarking wheat head detection methods. Plant Phenomics.

[23] Prajapati H.B., Shah J.P., Dabhi V.K. (2017). Detection and classification of rice plant diseases. Intell. Decis. Technol..

[24] Wu X., Zhan C., Lai Y.-K., Cheng M.-M., Yang J. (2019). 2019 IEEE/CVF Conference on Computer Vision and Pattern Recognition (CVPR).

[25] Cordts M., Omran M., Ramos S., Rehfeld T., Enzweiler M., Benenson R., Franke U., Roth S., Schiele B. (2016). 2016 IEEE Conference on Computer Vision and Pattern Recognition (CVPR).

[26] Russell B.C., Torralba A., Murphy K.P., Freeman W.T. (2008). LabelMe: a database and web-based tool for image annotation. Int. J. Comput. Vis..

[27] Wang H., Lyu S., Ren Y. (2021). Paddy rice imagery dataset for panicle segmentation. Agronomy.

[28] Madec S., Irfan K., Velumani K., Baret F., David E., Daubige G., Samatan L.B., Serouart M., Smith D., James C., Camacho F., Guo W., De Solan B., Chapman S.C., Weiss M. (2023). VegAnn, vegetation annotation of multi-crop RGB images acquired under diverse conditions for segmentation. Sci. Data.

[29] Scharr H., Minervini M., Fischbach A., Tsaftaris S. (2014).

[30] Haug S., Ostermann J., Agapito L., Bronstein M.M., Rother C. (2015). Computer Vision - ECCV 2014 Workshops.

[31] Mortensen A.K., Skovsen S., Karstoft H., Gislum R. (2019). 2019 IEEE/CVF Conference on Computer Vision and Pattern Recognition Workshops (CVPRW).

[32] Weyler J., Magistri F., Marks E., Chong Y.L., Sodano M., Roggiolani G., Chebrolu N., Stachniss C., Behley J. (2023). PhenoBench -- A large dataset and benchmarks for semantic image interpretation in the agricultural domain (arXiv:2306.04557). arXiv.

[33] Jia Z., Chen J., Xu X., Kheir J., Hu J., Xiao H., Peng S., Hu X.S., Chen D., Shi Y. (2023). The importance of resource awareness in artificial intelligence for healthcare. Nat. Mach. Intell..

[34] Tangseng P., Wu Z., Yamaguchi K. (2017). *Looking at outfit to parse clothing* (arXiv:1703.01386). arXiv.

[35] Long J., Shelhamer E., Darrell T. (2015). Fully convolutional networks for semantic segmentation (arXiv:1411.4038). arXiv.

[36] Shelhamer E., Long J., Darrell T. (2017). Fully convolutional networks for semantic segmentation. IEEE Trans. Pattern Anal. Mach. Intell..

[37] Zhao H., Shi J., Qi X., Wang X., Jia J. (2017).

[38] Chen L.-C., Zhu Y., Papandreou G., Schroff F., Adam H., Ferrari V., Hebert M., Sminchisescu C., Weiss Y. (2018). Computer Vision – ECCV 2018.

[39] Xie E., Wang W., Yu Z., Anandkumar A., Alvarez J.M., Luo P. (2021). SegFormer: simple and efficient design for semantic segmentation with transformers. Adv. Neural Inf. Process. Syst..

[40] Zhang W., Pang J., Chen K., Loy C.C. (2024). Proceedings of the 35th International Conference on Neural Information Processing Systems.

[41] Cheng B., Misra I., Schwing A.G., Kirillov A., Girdhar R. (2022).

[42] Chang T.-G., Wei Z.-W., Shi Z., Xiao Y., Zhao H., Chang S.-Q., Qu M., Song Q., Chen F., Miao F., Zhu X.-G. (2023). Bridging photosynthesis and crop yield formation with a mechanistic model of whole-plant carbon–nitrogen interaction. Silico Plants.

[43] Wang A., Zhang W., Wei X. (2019). A review on weed detection using ground-based machine vision and image processing techniques. Comput. Electron. Agric..

[44] Dosovitskiy A., Beyer L., Kolesnikov A., Weissenborn D., Zhai X., Unterthiner T., Dehghani M., Minderer M., Heigold G., Gelly S., Uszkoreit J., Houlsby N. (2020, October 2). International Conference on Learning Representations.

[45] He K., Zhang X., Ren S., Sun J. (2016). 2016 IEEE Conference on Computer Vision and Pattern Recognition (CVPR).

[46] Gao Y., Li Y., Jiang R., Zhan X., Lu H., Guo W., Yang W., Ding Y., Liu S. (2023). Enhancing green fraction estimation in rice and wheat crops: a self-supervised deep learning semantic segmentation approach. Plant Phenomics.

[47] Gao Y., Li L., Weiss M., Guo W., Shi M., Lu H., Jiang R., Ding Y., Nampally T., Rajalakshmi P., Baret F., Liu S. (2024). Bridging real and simulated data for cross-spatial- resolution vegetation segmentation with application to rice crops. ISPRS J. Photogrammetry Remote Sens..

[48] Li Y., Zhan X., Liu S., Lu H., Jiang R., Guo W., Chapman S., Ge Y., Solan B., Ding Y., Baret F. (2023). Self-supervised plant phenotyping by combining domain adaptation with 3D plant model simulations: application to wheat leaf counting at seedling stage. Plant Phenomics.

[49] Liu S., Martre P., Buis S., Abichou M., Andrieu B., Baret F. (2019). Estimation of plant and canopy architectural traits using the digital plant phenotyping Platform1 [OPEN]. Plant Physiol..

[50] Liu S., Baret F., Abichou M., Manceau L., Andrieu B., Weiss M., Martre P. (2021). Importance of the description of light interception in crop growth models. Plant Physiol..

[51] Oquab M., Darcet T., Moutakanni T., Vo H., Szafraniec M., Khalidov V., Fernandez P., Haziza D., Massa F., El-Nouby A., Assran M., Ballas N., Galuba W., Howes R., Huang P.-Y., Li S.-W., Misra I., Rabbat M., Sharma V., Bojanowski P. (2024). DINOv2: learning robust visual features without supervision (arXiv:2304.07193). arXiv.

