# Supplementary material for: Global rice multiclass segmentation dataset (RiceSEG): comprehensive and diverse high-resolution RGB-annotated images for the development and benchmarking of rice segmentation algorithms
Source: Plant Phenomics. 2025 Sep 4;7(3):100099. doi: 10.1016/j.plaphe.2025.100099 (PMC12710049; doi:10.1016/j.plaphe.2025.100099)
Supplement: Multimedia component 1 [file mmc1.docx]

**Plant Phenomics Supporting Information**

**Global Rice Multi-Class Segmentation Dataset (RiceSEG): A Comprehensive and Diverse High-Resolution RGB-Annotated Images for the Development and Benchmarking of Rice Segmentation Algorithms**

## Junchi Zhou^1^, Haozhou Wang^2^, Yoichiro Kato^2^, Tejasri Nampally^3^, P. Rajalakshmi^4^, M. Balram^5^, Keisuke Katsura^6^, Hao Lu^7^, Yue Mu^1^, Wanneng Yang^8^, Yangmingrui Gao^1^, Feng Xiao^1^, Hongtao Chen^1^, Yuhao Chen^1^, Wenjuan Li^9^, Jingwen Wang^10^, Fenghua Yu^11^, Jian Zhou^12^, Wensheng Wang^13^, Xiaochun Hu^14^, Yuanzhu Yang^14^, Yanfeng Ding^1^, Wei Guo^2, *^, Shouyang Liu^1, *^

## 1 Cross-site Generalization Experiment

## To verify that the overall conclusions are not dominated by the two largest contributing sites HUN and JL, all six baseline models were retrained after removing every image from these two sites (≈ 35 % of the annotated pixels) and reevaluated on the test set. A drop of only 0.5–1.6 percentage points and unchanged model ranking confirms robust cross-site generalization.

Table S1. Cross-site Generalization Experiment

| Model | mIoU (Full training set) | mIoU (Without HUN & JL) | Δ mIoU |
| --- | --- | --- | --- |
| FCN | 54.8 | 54.3 | −0.5 |
| PSPNet | 68.2 | 66.9 | −1.3 |
| DeepLabV3+ | 65.9 | 64.7 | −1.2 |
| SegFormer | 72.7 | 71.1 | −1.6 |
| KNet | 71.9 | 70.6 | −1.3 |
| Mask2Former | 74.7 | 73.9 | −0.8 |

## 2 Site-wise and Class-wise IoU for the Best Model (Mask2Former)

## Using the best-performing Mask2Former, we reported site-by-site mIoU and class-wise IoU. A few small sites show lower scores mainly because they contain very few test images, so a single error strongly affects the average, and because their class-pixel ratios are extremely unbalanced, making small classes easier to overlook or misclassify. Nevertheless, performance trends remain consistent across sites with very different class compositions, illumination conditions, and growth stages.

Table S2. Site-wise and Class-wise Experiment(“/” indicates that the class is absent in that site’s test images.)

| Site | mIou | background | green_veg | senescent_veg | panicle | weed | duckweed |
| --- | --- | --- | --- | --- | --- | --- | --- |
| GD | 53.19 | 47.37 | 85.8 | 39.76 | 67.92 | 25.1 | / |
| GX | 63.9 | 92.14 | 73.79 | 26.08 | / | / | / |
| HB | 78.1 | 77.76 | 90.81 | 64.13 | 79.53 | / | / |
| HLJ | 79.25 | 79.75 | 78.75 | / | / | / | / |
| HN | 57.82 | 90.07 | 87.77 | 30.67 | / | 41.58 | 39.04 |
| HUN | 54.2 | 36.83 | 88.64 | 45.1 | 82.91 | 17.7 | / |
| JL | 59.91 | 36.75 | 85.32 | 30.48 | 85.62 | / | 61.38 |
| JS_1 | 74.8 | 87.82 | 89.81 | 34.4 | 87.17 | / | 55.66 |
| JS_2 | 67.06 | 91.81 | 91.37 | 25.79 | / | / | 59.28 |
| JS_3 | 73.52 | 79.24 | 80.32 | 42.46 | 75.44 | 80.72 | 82.97 |
| JS_4 | 59.7 | 41.96 | 70.17 | 69.16 | 69.68 | 47.53 | / |
| JX | 75.45 | 87.88 | 63.02 | / | / | / | / |
| LN | 62.09 | 71.68 | 89.73 | 47.76 | 90.88 | 12.83 | 59.63 |
| TKO_1 | 63.7 | 95.19 | 95.77 | 70.73 | / | 36.56 | 20.25 |
| TKO_2 | 69.1 | 95.1 | 90.59 | 50.78 | 88.5 | 36.31 | 53.32 |
| TKO_3 | 73.73 | 66.41 | 93.3 | 57.4 | 77.8 | / | / |
| Telangana | 63.55 | 89.96 | 85.55 | 22.57 | 86.91 | 32.76 | / |
| Laguna | 52.48 | 90.17 | 88.1 | 15.69 | / | 15.96 | / |
| Kilimanjaro | 56.45 | 45.65 | 60.1 | 49.68 | 70.39 | / | / |

## 3 Global range distribution and composition of the datasets


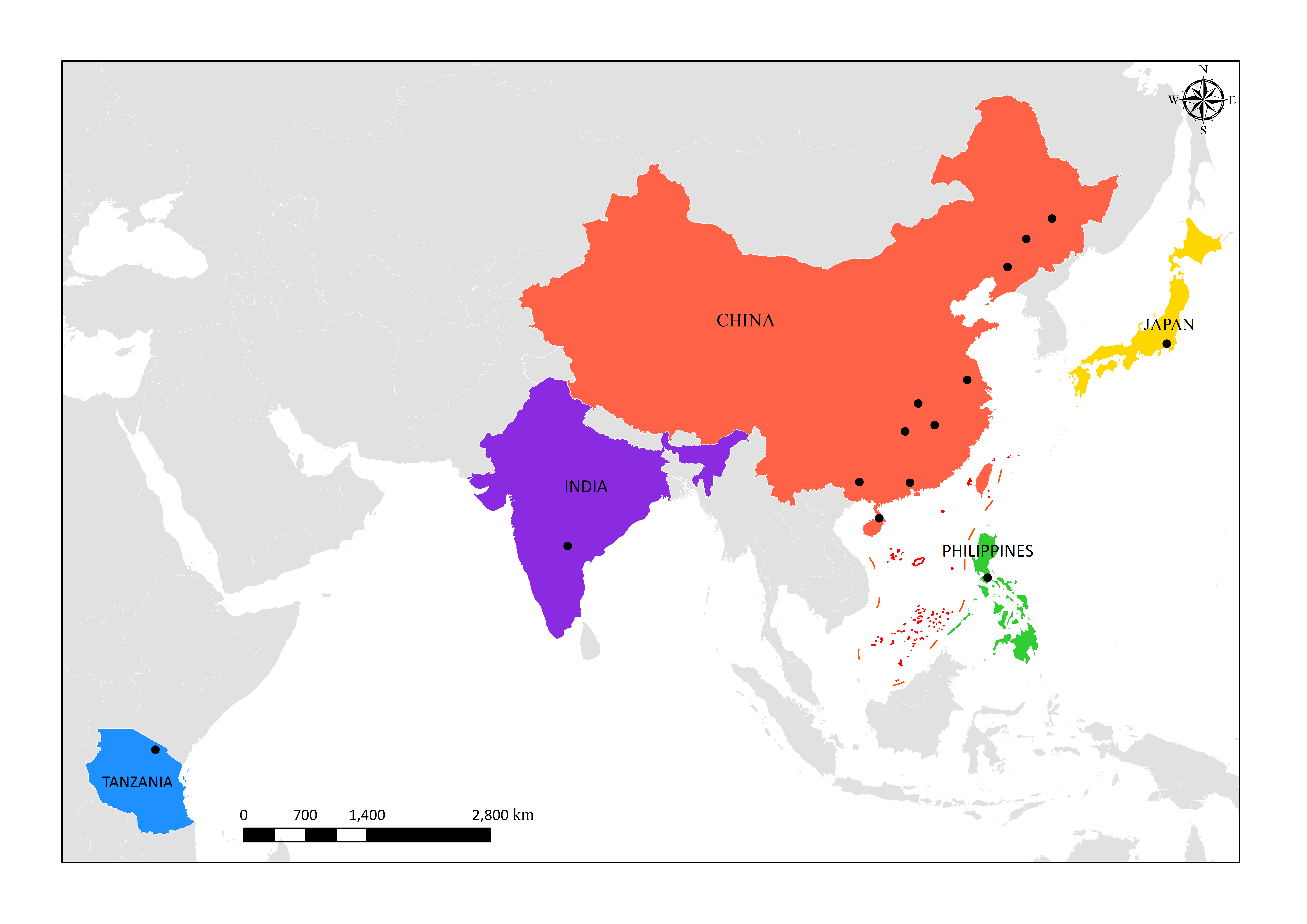


## 4 Reproducibility Further source information and all original images can be found and accessed at www.global-rice.com.
